# Structure and Functional Characteristics of Gelatin Extracted from Grass Carp (*Ctenopharyngodon idella*) By-Products

**DOI:** 10.3390/foods14234086

**Published:** 2025-11-28

**Authors:** Jiandong Shen, Lijun Fu, Bijiang Zhong, Wenshui Xia, Yanshun Xu

**Affiliations:** 1 Fujian Provincial Key Laboratory of Ecological Impacts and Treatment Technologies for Emerging Contaminants, Key Laboratory of Ecological Environment and Information Atlas, College of Environmental and Biological Engineering, Putian University, Putian 351100, China; lijun_fu@sina.com; 2 Office of Science and Research, Putian University, Putian 351100, China; bjzhongzg@163.com; 3 State Key Laboratory of Food Science and Resources, School of Food Science and Technology, Jiangnan University, Wuxi 214122, China; xiaws@jiangnan.edu.cn

**Keywords:** grass carp, by-products, gelatin, structure, functional property

## Abstract

The recycling of by-products from fish processing procedures has recently been attracting increased attention. In this study, three types of gelatin were isolated from grass carp skin, bone, and scales, named SKG, BG, and SCG, respectively, and their structural and functional characteristics were investigated. Compared with BG and SCG, SKG exhibited the highest extraction yield (18.30 ± 0.24%) and protein content (90.12 ± 0.21%) and the lowest ash content (1.50 ± 0.08%). Electrophoresis analysis revealed that SKG contained more α chains than BG and SCG. Fourier transform infrared spectra showed that the absorption peaks of gelatin were mainly positioned in amide band regions, whereas some of the triple helix structure was lost. More than 85% solubility was observed in all gelatin types with pH 3–10. Meanwhile, there was a higher gel strength in SKG (288.2 g) than in BG (270.2 g) and SCG (245.1 g). Furthermore, the water or oil absorption and emulsifying characteristics of SKG were also better than those of BG and SCG. The differences in functional properties between gelatin types appear to be related to protein distribution and composition. All the results indicate that grass carp skin is a material with the potential to extract gelatin with a higher yield and gel strength and better functional characteristics compared with bone and scales.

## 1. Introduction

Gelatin is a series of polypeptides prepared using a large number of collagen molecules through biological degradation or heat denaturation [1]. Through the breaking of hydrogen or covalent bonds and the degradation of their triple helix region, collagen molecules can be transformed into different soluble gelatin molecules. These proteins present unique physicochemical properties, indicating their potential use as a thickener, emulsifier, gelling agent, and foaming agent, and are widely used in the food industry [2]. With demand growing, the output of gelatin products currently exceeds more than USD 6.5 billion annually [3]. At present, gelatin products are mainly extracted from mammalian by-products (skin, bone, and tendons). However, due to limiting factors such as zoonosis diseases and religious reasons, the utilization of gelatin recovered from porcine and bovine sources is restricted [4]. Furthermore, chemical reagents must be applied to remove the fat and pigment of by-products during the extraction process, causing a series of environmental problems. These issues have led to an increased focus on obtaining other potential types of gelatin as a substitution for mammalian gelatin.

In recent years, gelatin has been extracted from many fish species, such as the spotted golden goatfish [5], tilapia [6], and grass carp [7]. Fish gelatin has revealed excellent properties, such as biocompatibility, low allergenicity, and emulsifying activities [8]. It has also shown admirable performance in maintaining the stability of oils during emulsion preparations [9]. These functional characteristics of gelatin products are related to protein distribution and composition [10]. Additionally, gelatin quality was influenced by the fish type, fish age and size, and fish tissue type, as well as the processing method. The gelatin extraction yields revealed that the pepsin enzyme method was more effective in extracting gelatin from Tilapia skin than the acetic acid method and the heating method [6]. The gel strength of bigeye snapper (*Priacanthus macracanthus*) gelatin was higher than that of the purple-spotted bigeye (*Priacanthus tayenus*) with the same extraction method [11].

The output of freshwater fish reached approximately 28.0 million tons in 2023, which made up 80% of freshwater products in China [12]. In recent years, with the growing demand for fish fillets and surimi, the proportion of by-products from freshwater fish processing has increased. Approximately 60% of fish by-products (bone, viscera, skin, and scale) are produced during these processing procedures. Previous studies found abundant proteins in these by-products, especially collagen in fish tissues (bone, skin, and scale) [8]. However, because of technological restrictions and consumer concepts, fish by-products are commonly used as pet food or discarded as waste. Recently, fish by-products have been identified as good sources for extracting gelatin, contributing to solving waste disposal issues and creating value-added products [1]. The differences among parts of grass carp used for gelatin extraction could have an impact on the composition and properties of the gelatin. However, to the best of our knowledge, the structural and functional properties of gelatin extracted from different grass carp by-products have not been fully studied.

In this work, gelatin was prepared from grass carp skin, bone, and scale. The structural characteristics of gelatin were confirmed via electrophoretic analysis, Fourier transform infrared spectrum analysis, and scanning electron microscope observation. Functional characteristics of different gelatin such as gel strength, protein solubility, water or oil absorption, and emulsifying properties were also studied. Our research is beneficial as it investigates the relationship between the structure and functional characteristics of gelatin from different tissues and provides critical information to guide the efficient utilization of by-products derived from freshwater fish.

## 2. Materials and Methods

### 2.1. Fish Gelatin

Grass carp (3.20 ± 0.15 kg, *n* = 20) was obtained from a fish supermarket (Putian, Fujian Province, China). Fish were slaughtered by a trained worker in the laboratory of Putian University, and fish by-products (skin, bone, and scale) were cleaned and then cut into standard-sized samples of 2.0 ± 0.1 cm in length. Each by-product was mixed with a 6-fold volume of 1.0% NaOH (*w*/*v*). Gelatin was prepared as described by Tan et al. [13], with some modifications. To remove non-collagen and fat, the alkaline solution was stirred and replaced each 6 h. After the fourth alkaline solution washing, the insoluble portion was rinsed with distilled water. Subsequently, the insoluble substance and 0.1% HCl were mixed in a ratio of 1:6 (*w*/*v*) and soaked for 3 h. After acid treatment, residual samples were washed until reaching a neutral pH and mixed with a 6-fold volume of distilled water (*w*/*v*). After being extracted at 70 °C for 2 h, obtained samples were rapidly cooled to room temperature, followed by centrifuging at 10,000× *g* for 10 min. The obtained supernatant was adequately dialyzed against distilled water and then dried at 45 °C, and the obtained samples were treated as gelatin for the purpose of further experiments.

### 2.2. Extraction Yield of Gelatin

The following equation was adopted to measure the extraction yield of gelatin [14]: extraction yield (%) = m/S × 100. The m represents the gelatin dry weight, and S represents the grass carp skin, bone, or scale dry weight.

### 2.3. Composition of Gelatin

The composition (ash, protein, fat, and moisture) of gelatin was analyzed based on our previously described procedures [15].

### 2.4. Electrophoretic Analysis

The molecular mass of gelatin was analyzed via gel electrophoresis analysis using 8% resolving gel [16]. Gelatin (30.0 μg) was loaded per well, and protein markers were applied to evaluate the molecule mass. After electrophoresis at 10 mA, the obtained gels were placed in Coomassie Blue R-250 solution and then destained in a 30% methyl alcohol and 10% acetic acid solution. Photographs of the final gels were observed through a gel imaging system (ChemiDoc MP Gel Imaging system, Bio-Rad Company, Hercules, CA, USA), with all protein bands of gels appearing against a clear background.

### 2.5. Fourier Transform Infrared Spectrum Analysis

The spectra of gelatin were recorded using a spectrometer (Tensor 27, Bruker, Mannheim, Germany) with Fourier transform, as introduced by Chen et al. [17]. Measurements were carried out at room temperature and with a wavelength coverage of 500–4000 cm^−1^.

### 2.6. Scanning Electron Microscopy (SEM) Analysis

The morphological characteristics of gelatin were measured using an SU8100 SEM (Hitachi High-Tech Instruments Co., Ltd., Tokyo, Japan) [18]. Before determination, gelatin was fixed on a metal platform and coated with Pd. The measurements were performed at 15.0 kV, and magnifications were 100× and 500×.

### 2.7. Gel Strength Analysis

The gel strength of gelatin was analyzed according to the procedures of Nikoo et al. [19], using a CT3-10K texture analyzer (Brookfield Corporation, New York, NY, USA) with a diameter of 12.7 mm and a flat-bottomed plunger. A 6.67% gelatin solution (*w*/*v*) was obtained by mixing 3.0 g of gelatin and 44.9 mL of distilled water, followed by stirring for 30 min, and maintained at 10 °C for 18 h. The gel strength was defined as the maximum value of reaction force when the flat-bottomed plunger had penetrated gels up to 4.0 mm.

### 2.8. Determination of Solubility

To investigate the influence of pH on protein solubility, gelatin (10.0 mg) and distilled water (8.0 mL) were mixed in centrifuge tubes and adjusted to pH 3–10 with HCl or NaOH solution, respectively [15]. The mixed solution was made up to 10.0 mL with distilled water, followed by incubation at room temperature for 30 min and centrifugation at 4000× *g* for 10 min. The proteins in the supernatant of the final samples and in gelatin were measured using the Kjeldahl method. Gelatin solubility was calculated using the following equation: solubility (%) = T1/T × 100, where T1 represents the protein in the supernatant of final samples and T represents the total protein in gelatin.

### 2.9. Water-Holding Capacity (WHC) and Oil-Holding Capacity (OHC)

The WHC of gelatin was assessed using the procedures of Alahmad et al. [20], with several changes. Test samples were prepared with gelatin (1.0 g) mixed with distilled water (10.0 mL) and maintained at 37 °C for 60 min. Following centrifugation at 450× *g* for 5 min, the precipitate was collected. The WHC was presented as the amount of gelatin (g) absorbed of water (mL).

For the OHC of gelatin, we referred to the procedures of Jellouli et al. [21]. Samples were prepared comprising gelatin (0.2 g) mixed with soybean salad oil (6.0 mL) in centrifuge tubes. When this step was complete, the mixed samples were maintained at 37 °C for 1 h. After centrifugation at 3000× *g* for 20 min, the absorption of oil was measured based on volume variance. The OHC is presented as the amount of gelatin (g) absorbed by oil (mL).

### 2.10. Emulsifying Characteristics

The emulsion activity index (EAI) and emulsion stability index (ESI) were determined based on the methods of Zheng et al. [22], with some modification. First, 0.05 g of samples and 50.0 mL of corn oil were mixed at the speed of 6000 rpm for 1 min. At the reaction times of 0 min and 10 min, a volume of 1.0 mL was taken from the bottom and adjusted to 50.0 mL by adding sodium dodecyl sulphate (0.1%, *w*/*v*). The absorbance of final samples was measured at a wavelength of 500 nm. EAI and ESI are presented based on the following equations: EAI (m_2_/g) = 2 × k × A_0_/0.25/M and ESI (min) = A_0_ × t/(A_0_−A_10_). A_0_ and A_10_ are the values of the final solution at 0 min and 10 min, respectively. The k is 2.303, M represents the weight of gelatin, and t represents 10 min.

### 2.11. Statistical Analysis

All experiments were implemented in triplicate. Statistical analyses were conducted using SPSS 21.0 software (SPSS Inc., Chicago, IL, USA). All results are shown as means ± standard deviations, and one-way analysis of variance and Duncan’s multiple range test were applied to calculate the significance of data; a significance level of *p* < 0.05 was chosen to determine statistically significant differences.

## 3. Results and Discussion

### 3.1. Extraction Yield and Chemical Composition of Gelatin

The extraction yields of gelatin extracted from grass carp skin, bone, and scale are illustrated in Table 1. An obvious difference in extraction yield can be observed among skin gelatin (SKG), bone gelatin (BG), and scale gelatin (SCG) (*p* < 0.05). The value for the extraction yield for SKG (18.30 ± 0.24%) was significantly higher than those for BG (6.95 ± 0.13%) and SCG (8.39 ± 0.32%) (*p* < 0.05). Variations in extraction yields might be attributed to different factors, such as the amount of collagen in fish [1], the composition of connective tissues [23], and the extraction conditions [24]. Compared with bone and scale, more collagen was found in large yellow croaker (*Larimichthys crocea*) skin [25]. According to Purslow (2014), crosslinks are considered an important factor in maintaining structural stability in collagen molecules [23]. A lower content of crosslinks is found in fish skin than in bone and scale [26]. Therefore, gelatin might be more easily released from grass carp skin than bone and scale using the same heating treatment.

The composition (moisture, protein, fat, and ash) of SKG, BG, and SCG is also displayed in Table 1. All obtained gelatin revealed a relative higher concentration of protein and a lower amount of moisture and fat. The moisture content was 9.25 ± 0.13%, 9.84 ± 0.11%, and 9.42 ± 0.06% for SKG, BG, and SCG, respectively. The moisture of the three gelatin samples was below the recommended value (15%) for edible gelatin [24]. For protein content, the highest concentration was 90.12 ± 0.21% in SKG, whereas the lowest content was 82.62 ± 0.15% in SCG. Abundant minerals (calcium and phosphorus) were found in fish bone and scale, and they could be co-solubilized with gelatin dissolution [11]. The ash content of SKG, BG, and SCG ranged from 1.50% to 9.20%. The ash content of the obtained BG and SCG was generally higher than that of SKG. This might be due to the release of minerals from the bone and scale during the extraction process [11]. Moreover, SKG had a low ash content (1.51%), which was below the recommended maximum of 2.6%. On the contrary, a higher ash content was found in BG (9.20%) and SCG (8.94%). Calcium is the main mineral in fish bone and scale and presents as hydroxyapatite [11]. Hydroxyapatite maintains the structural stability of fish bone and scale and makes it difficult to dissolve gelatin, which could explain the low extraction yield of BG and SCG. BG and SCG cannot be directly utilized as food ingredients, but they may be used as a calcium supplement in food products.

### 3.2. Protein Patterns in Gelatin

**Figure 1:**
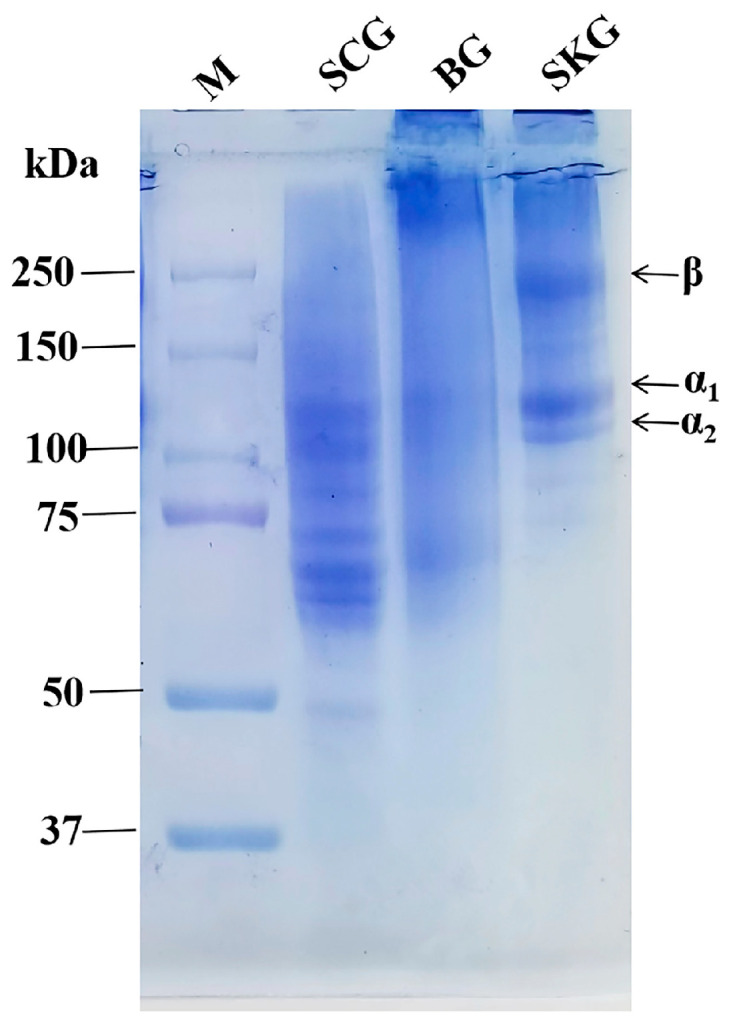
SDS-PAGE patterns of gelatin extracted from grass carp skin, bone, and scale. M, protein maker; SKG, skin gelatin; BG, bone gelatin; SCG, scale gelatin.

The protein patterns of SKG, BG, and SCG with the same extraction method are analyzed in Figure 1. By comparing protein markers via SDS-PAGE, it was observed that gelatin consisted of proteins of varying molecular weight (50 kDa to 300 kDa). These different molecular weights of gelatin are due to the random conversion of collagen molecules to smaller peptide molecules via the degradation of inter-chain crosslinks and peptide linkages during heating treatment [19]. We also found that SKG showed a more intact molecular weight distribution of collagen molecules than BG and SCG. In all gelatin samples, α chains were the major constituents in electrophoretic analysis, with the molecular weight of 125 kDa (α_1_) and 113 kDa (α_2_). Moreover, there was a higher concentration of β chains and polymers in SKG compared with BG and SCG. Obviously, the gelatin obtained from the three grass carp by-products was of varying molecular weight and composition. Crosslinks were reported to maintain the structural stability of collagen molecules [23]. Due to the higher number of crosslinks in bone and scale, gelatin could not be effectively extracted, and a lower concentration of gelatin was observed for BG and SCG via SDS-PAGE. Thus, we can infer that a crosslinked structure makes it more difficult to extract α-chain dimers and trimers from collagen via heating treatment [27]. These results are consistent with the extraction yields of gelatin given in Table 1, in which more proteins were prepared from grass carp skin than from other tissues. In addition, the ratio of low-molecule proteins was higher in the protein profile of BG and SCG. The low-molecular-weight bands might be the degraded products of α and β chains from the thermal process. Meanwhile, the yield and gelatin composition were affected by the extracting temperature and time. A high extraction temperature (75 °C) leads to a higher extracted yield of gelatin than a low extraction temperature (45, 55, and 65 °C), but it might degrade proteins and lower their quality [13]. Furthermore, our extraction conditions (temperature and time) should be further optimized to obtain high-quality gelatin.

### 3.3. Fourier Transform Infrared Spectra of Gelatin

**Figure 2:**
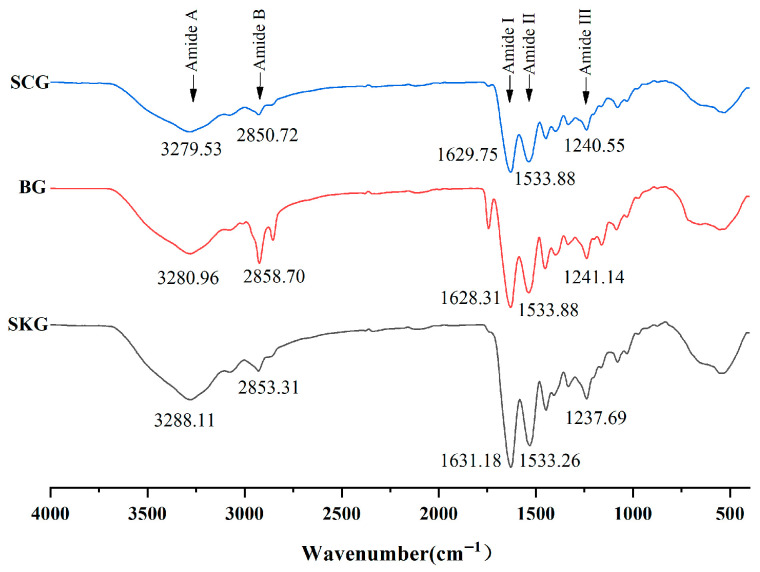
FTIR spectra of gelatin extracted from grass carp skin, bone, and scale. SKG, skin gelatin; BG, bone gelatin; SCG, scale gelatin.

FTIR spectroscopy is widely used to monitor the structural characteristics of proteins. Five amide bonds (I, II, III, A, and B) of collagen molecule had special peaks, which were associated with the protein order and the integrity of the triple helix region [15]. The FTIR spectra of SKG, BG, and SCG are displayed in Figure 2. The absorption of amide I bands of SKG, BG, and SCG was 1631.18 cm^−1^, 1628.31 cm^−1^, and 1629.75 cm^−1^, respectively. The peak of amide I represented the C=O stretching vibration, combined with contributions from CCN deformation and CN stretching. The shifting of amide I to a lower frequency indicated that the coil structural order of collagen was changed [28]. Lower peaks of amide I in BG and SCG suggested fewer inter-molecular crosslinks. For amide II bands, the absorption peaks of all gelatin types revealed similar spectra. Furthermore, the rate of amide III/1450 cm^−1^ was not equal to 1.0, suggesting that part of the helical structure remained in gelatin products. Consistent with the data in Figure 1, α chains were found in all samples. The characteristic absorption wavelengths of amide A for BG and SCG (3280.96 cm^−1^ and 3279.55 cm^−1^) were lower than for SKG (3288.11 cm^−1^). A lower frequency of amide A suggested that the N-H group of shorter chain peptides participated in hydrogen bonds. The lower amide A value was generally considered to represent the breakdown of collagen and a decrease in free amino groups between gelatin and other proteins.

### 3.4. Microstructures of Gelatin

**Figure 3:**
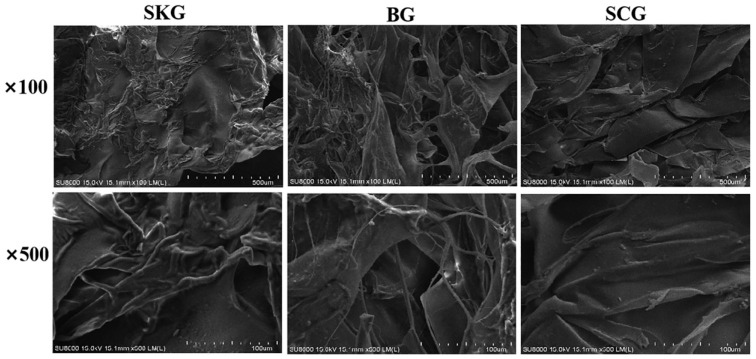
Microstructures of gelatin gel from grass carp skin, bone, and scale. SKG, skin gelatin; BG, bone gelatin; SCG, scale gelatin. Magnification: 100×, 500×.

As illustrated in Figure 3, the microstructural properties of gelatin isolated from different grass carp tissues (skin, bone, and scale) were detected via scanning electron microscopy. When the magnification was 100× and 500×, a tightly parallel-aligned structure was observed in SKG. Conversely, BG had a looser network with thinner strands and larger spaces. It is known that the gel network is controlled by the molecular distribution of gelatin and pretreatment conditions [29] and is related to the molecular distribution of α, β, and γ-chains. As a result, high-molecular-weight gelatin easily forms connection regions with ordered gel structures. Similarly, higher-molecular-weight α chains more easily gathered, to create a strong network with a higher gel strength [28]. Therefore, with a higher molecular weight of chains, SKG could form more tightly parallel-aligned gels than BG and SCG. These results also suggest that gelatin from fish tissues with different molecule distribution could show different arrangement modes in the network. Furthermore, the gelatin extraction process should be optimized to obtain intact α and β chains.

### 3.5. Solubility and Gel Strength of Gelatin

**Figure 4:**
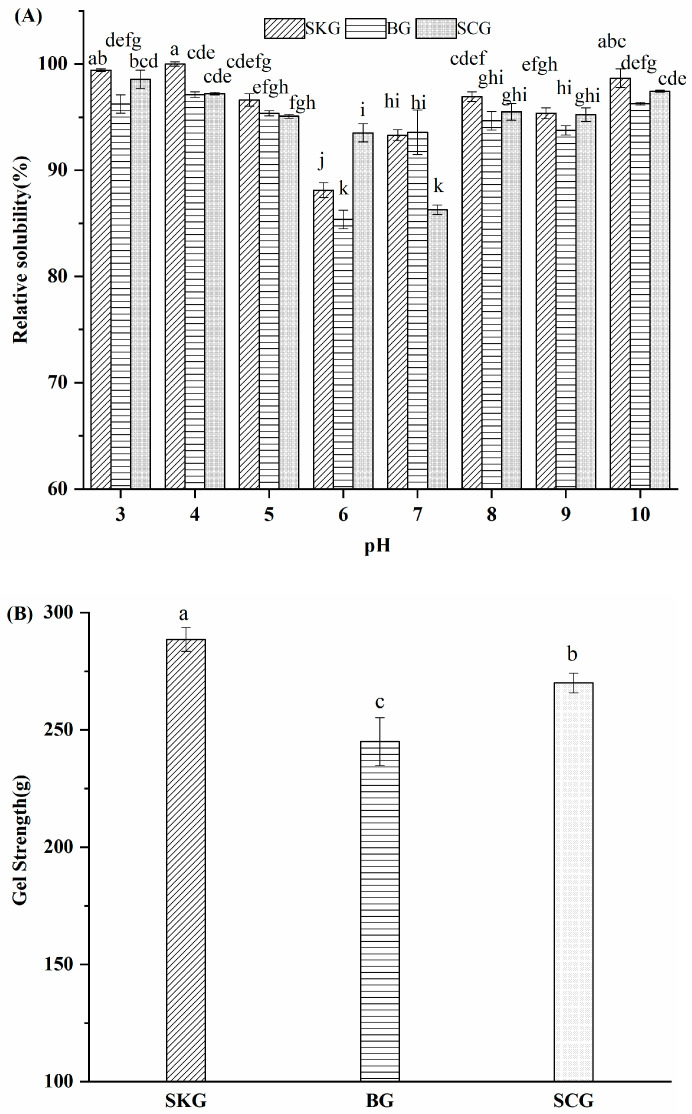
Solubility, as influenced by pH (**A**) and gel strength (**B**), of gelatin extracted from grass carp skin, bone, and scale. SKG, skin gelatin; BG, bone gelatin; SCG, scale gelatin. Points with different letters are significantly different (*p* < 0.05).

Solubility is a critical functional feature in the application of gelatin in the food industry. The relative solubility of SKG, BG, and SCG was analyzed at different pH values. As shown in Figure 4A, the relative solubility of gelatin was higher than 85% with pH 3–10. The highest values were observed for SKG, indicating that it contained abundant hydrophilic domains. Higher hydrophilic domains at the surface of gelatin mean that it can easily interact with more water [28]. The highest solubility of gelatin was found when the pH was below 5 and above 6, suggesting that it had a wide pH range. The lowest solubility for SKG and BG was at pH 6.0, and for SCG, it was at pH 7. Sac-leaw previously reported that the lowest solubility of gelatin extracted from defatted seabass skin was found at pH 6 [30]. Generally, the isoelectric points of type-A gelatin were between pH 6 and pH 9. These results indicate that SKG, BG, and SCG might belong to type-A gelatin. The minimum solubility value was obtained at the isoelectric point of gelatin, which might be because of aggregation and precipitation via hydrophobic interactions. The difference in the isoelectric point of gelatin might be related to the raw material, molecular weight of gelatin, extraction method, or amino acid composition.

The gel strength of SKG, BG, and SCG is shown in Figure 4B. Gelatin gels prepared from SKG revealed higher gel strength (288.2 g) than those from BG (270.2 g) and SCG (245.1 g). Gel strength has been found to be different in gelatin of other types from other fishes, including tilapia skin (144 g) [6] and seabass swim bladder (204.3 g) gelatin [4]. Moreover, the gel strength of our three obtained gelatin samples was higher than that of commercial porcine gelatin (224.3 g), indicating that our fish gelatin has potential applications [31]. Gel strength is governed by gelatin molecular weight, and larger molecules can produce stronger strands to form gel networks [28]. The value of gel strength was in accordance with the protein band intensity observed via SDS-PAGE (Figure 1). SKG revealed higher band intensity of α and β chains than BG and SCG. Therefore, these results indicated that gelatin with a higher ratio of α and β chains revealed better gelling quality. Furthermore, gel strength was also related to the concentration of amino acids. Hydroxyproline, the specific amino acid in collagen molecules, serves a critical function in protecting triple-helical regions. These results are in line with the secondary structure detected via FTIR (Figure 2) and the band intensity of α-chains (Figure 1). Therefore, the differences in gel strength in gelatin from different grass carp by-products might be related to their molecular weight distribution and amino acid composition.

### 3.6. Water-Holding Capacity and Oil-Holding Capacity

The WHC and OHC are related to texture, as they connect water and oil. As observed in Table 2, the WHC and OHC of SKG, BG, and SCG were measured. All gelatin types had a relative higher OHC compared with WHC. This result suggests that more hydrophobic amino acids than hydrophilic amino acids were present at the surface of the gelatin. SKG exhibited a better OHC than BG and SCG, indicating it contained a higher degree of exposure of hydrophobic residues. Similarly, Jellouli reported a higher amount of hydrophobic amino acids (tyrosine, isolecucine, and valine) in gelatin from grey triggerfish (*Balistes capriscus*), leading to a higher OHC than that observed in bovine-hide gelatin [21].

### 3.7. Emulsifying Capacity and Stability of Gelatin

Emulsifying properties refer to the stability of the area of the oil–water interface. Gelatin is commonly used as an emulsifier because of its emulsion activity index (EAI) and emulsion stability index (ESI). At the same concentration, the EAI of SKG was significantly higher compared to that of BG and SCG (Table 2). The value of EAI was influenced by different factors, including the amino acids, intrinsic properties, and conformation of proteins. It was noticeable that a high solubility level in gelatin increased the emulsification efficiency during the dispersing stages. Meanwhile, it was also found that SKG had a better ESI than that of BG and SCG (Table 2). This could be explained by the fact that the larger molecules in SKG more effectively maintained the stability of the gelatin film at the interface. All these results support SKG as a potential emulsifier in the food industry.

## 4. Conclusions

Gelatin was prepared from grass carp by-products (skin, bone, and scale), with α chains as the major components. Compared with bone gelatin (BG) and scale gelatin (SCG), the highest extraction yield and protein content were observed for skin gelatin (SKG). Meanwhile, all gelatin types showed excellent solubility across wide pH ranges. The functional properties, including gel strength, water or oil absorption, the emulsion activity index (EAI), and the emulsion stability index (ESI), were superior in SKG to those of BG and SCG. The difference in functional properties between gelatin types appeared to be related to molecular weight distribution and protein composition. This study supports grass carp skin as a potential material from which to extract gelatin with a higher yield and excellent functional characteristics. Further investigations are required to obtain further insights into optimizing the extracting conditions of grass carp skin gelatin, as well as into its potential applications in food products.

## Figures and Tables

**Table 1:** Extraction yield (dry weight) and gel colour of gelatin extracted from grass carp skin, bone, and scale.

		SKG	BG	SCG
Yield (%)		18.30 ± 0.24 a	6.95 ± 0.13 c	8.39 ± 0.32 b
Chemical composition (%)	Moisture	9.25 ± 0.13 b	9.84 ± 0.11 a	9.42 ± 0.06 ab
	Protein	90.12 ± 0.21 a	84.89 ± 0.30 b	82.62 ± 0.15 c
	Fat	0.23 ± 0.02 a	0.02 ± 0.01 c	0.03 ± 0.01 b
	Ash	1.50 ± 0.08 b	9.20 ± 0.14 a	8.94 ± 0.05 a

Values are expressed as means ± standard error (*n* = 3). Points with different letters are significantly different (*p* < 0.05). SKG, skin gelatin; BG, bone gelatin; SCG, scale gelatin.

**Table 2:** Water-holding capacity, oil-holding capacity, emulsion activity index (EAI), and emulsion stability index (ESI) of gelatin from grass carp.

	SKG	BG	SCG
Water-holding capacity	1.59 ± 0.05 b	1.53 ± 0.10 b	2.01 ± 0.11 a
Oil-holding capacity	5.54 ± 0.15 a	5.21 ± 0.08 a	4.18 ± 0.10 b
EAI (m^2^/g)	35.31 ± 1.21 a	26.87 ± 0.86 b	31.45 ± 0.58 a
ESI (min)	42.15 ± 0.23 a	38.24 ± 0.35 b	40.21 ± 0.36 b

Values are expressed as means ± standard error (n = 3). Points with different letters are significantly different (*p* < 0.05). SKG, skin gelatin; BG, bone gelatin; SCG, scale gelatin.

## Data Availability

The original contribution presented in this study are included in the article. Further inquiries can be directed to the corresponding author.
